# Outcomes of Elderly Patients Hospitalized with the SARS-CoV-2 Omicron B.1.1.529 Variant: A Systematic Review

**DOI:** 10.3390/ijerph20032150

**Published:** 2023-01-25

**Authors:** Roxana Manuela Fericean, Cristian Oancea, Akash Reddy Reddyreddy, Ovidiu Rosca, Felix Bratosin, Vlad Bloanca, Cosmin Citu, Satish Alambaram, Neeharika Gayatri Vasamsetti, Catalin Dumitru

**Affiliations:** 1 Department XIII, Discipline of Infectious Diseases, “Victor Babes” University of Medicine and Pharmacy Timisoara, Eftimie Murgu Square 2, 300041 Timisoara, Romania; 2 Doctoral School, “Victor Babes” University of Medicine and Pharmacy Timisoara, Eftimie Murgu Square 2, 300041 Timisoara, Romania; 3 Center for Research and Innovation in Precision Medicine of Respiratory Diseases, “Victor Babes” University of Medicine and Pharmacy Timisoara, Eftimie Murgu Square 2, 300041 Timisoara, Romania; 4 School of General Medicine, Bhaskar Medical College, Amdapur Road 156-162, Hyderabad 500075, India; 5 Department of Plastic Surgery, “Victor Babes” University of Medicine and Pharmacy Timisoara, Eftimie Murgu Square 2, 300041 Timisoara, Romania; 6 Department of Obstetrics and Gynecology, “Victor Babes” University of Medicine and Pharmacy Timisoara, Eftimie Murgu Square 2, 300041 Timisoara, Romania; 7 Faculty of General Medicine, Kaloji Narayana Rao University of Health Sciences, Nizampura, Warangal 506007, India

**Keywords:** COVID-19, SARS-CoV-2 infection, elderly patients, Omicron variant

## Abstract

The Omicron (B.1.1.529) variant of the severe acute respiratory syndrome Coronavirus 2 (SARS-CoV-2) had an increased rate of spreading among the general population. Although this virus mutation resulted in milder symptoms, those on the vulnerable side of the population are still in danger of developing severe symptoms. Thus, this systematic review focused on identifying the clinical outcomes of older age patients (>65) that are hospitalized with the SARS-CoV-2 Omicron variant. The research was conducted using four electronic databases (PubMed, Scopus, Web of Science, and ProQuest Central), with a search query in December 2022 that comprised the duration of the COVID-19 pandemic. The inclusion criteria comprised (1) a population of patients older than 65 years, (2) a history of hospitalization for SARS-CoV-2 infection, and (3) infection with Omicron B.1.1.529 variant. The initial search generated 295 articles, out of which six were included in the systematic review, and a total of 7398 patients. The main findings were that when looking at the elderly population, the mortality and hospitalization rates remained high. This is because older people are more vulnerable and have more comorbidities that interfere with the virus’s progress. However, there is inconsistency in mortality rates, since the data reported by the included studies had different selection criteria based on the severity of the COVID-19 infection. Although no statistically significant differences were found between the unvaccinated and vaccinated groups, patients who got booster doses of vaccination had a lower likelihood of developing severe symptoms, serving as a protective factor for this population.

## 1. Introduction

Since it emerged, the Coronavirus disease (COVID-19) has been given the highest priority on a global scale in the search for appropriate treatment, considering the colossal impact that it has had on the population. As of 2022, the World Health Organization (WHO) estimates 643,875,406 cases of people infected with the virus and 6,630,082 deaths [1]. The severe acute respiratory syndrome Coronavirus 2 (SARS-CoV-2), which is responsible for causing the infectious disease known as COVID-19, underwent multiple mutations over the course of three years, the most common of which was known as the Alpha, Beta, and Gamma variants, with the Delta and Omicron variants being more contagious than the other three [2]. Because COVID-19 is an RNA virus, it possesses the ability to undergo mutations as it duplicates. Because of this, the more times the genome replicates, the more variants are produced as the virus evolves, making it difficult to inhibit the virus’s ability to spread [2,3]. 

Although a number of studies point out that the Omicron variant has a less severe impact on populations than the Delta version does, it has multiple mutations, thus enabling it to spread faster and infect a larger population, causing symptoms mostly related to the upper respiratory tract [4]. The main problem that arises is linked to the effects that this type of mutation has on the more vulnerable population, which is more susceptible to its effects [4,5]. It has been found that hospitalized patients, who are a more vulnerable population, have a lower risk of developing severe symptoms if they have the Omicron variant. However, the risks that this variant implies are strongly connected to advanced age as well as other comorbidities such as heart conditions, metabolic disorders, chronic pulmonary disease, and obesity [5,6].

Recent systematic reviews and meta-analyses have stated that the elderly population represents the vulnerable sector of the population in the Omicron wave, having the lowest proportion of non-severe disease and asymptomatic infection [7,8]. One meta-analysis underlines the negative effect that older age and an immunocompromised system have on the immune response to COVID-19 vaccines, decreasing their effectiveness rates [7]. The main solution found up to this point is linked to an increased dosage of vaccines, as the two-dose scheme seems to be insufficient in this segment of the population [7,8,9]. According to the findings of one systematic review, giving a vulnerable population a third booster dose of vaccination considerably reduced the risk of hospitalization and prevented the development of severe symptoms [8]. Even though the vaccine’s efficacy may be lower for the Omicron form, especially in the vulnerable sector, it still has a high efficacy of over 70% against severe disease, according to the findings of another systematic review, which did not find differences across the types of vaccines used [9]. 

It would appear that the progression of the virus is not affected by geographic location, with each country exhibiting the same characteristics, risk factors, and protective factors. For instance, a study that was conducted in Sweden [10] found that the risk of severe disease was lower for unvaccinated people during the Omicron wave in comparison to the Delta wave, which was more aggressive. However, the risk remained high for the vulnerable population, which is comprised of elderly people and people who have multiple medical conditions [10]. Another study that was conducted in the United States came to the conclusion that the vaccinated cohort that required hospitalization consisted of elderly people who had a greater chance of having multiple medical conditions, but a third dose was found to reduce the rate of hospitalization [11]. In a nursing home in Serbia, it has been observed that although the rapid spreading of the virus could not be stopped, vaccines were a successful aid in preventing severe symptoms and reducing mortality rates [12]. 

Although the existing body of research literature indicates the requirement of a booster dose of vaccine for the older age segment of the population [7,8,9], there are a number of obstacles that exist that have the potential to limit this process. For the vulnerable population, it has been found that the elderly were reluctant to receive a booster vaccine for the following reasons: they lacked information about how the vaccine interferes with other comorbidities; they were concerned about its safeness; they had movement limitations; or they believed booster shots were unnecessary [13]. It is necessary to find a solution to this issue taking into consideration the emerging literature that calls attention to the requirement of increasing vaccine dosages in order for the treatment to be effective for the more susceptible members of the population [7,8,9,13]. In addition, a study that was carried out in China came to the conclusion that for hospitalized patients who carried the Omicron variant, older age made it more difficult for them to recover from the virus, and along with a depressive status, it constituted a risk factor that could lead to an Omicron re-positivity [14]. One expert review highlights the need for studies that focus on a vulnerable population, such as people that have an older age, comorbidities, or are immunocompromised, as this segment of the population has the highest risk of developing severe outcomes [15]. 

Taking into consideration the current state of the vulnerable population facing the COVID-19 Omicron virus, for which the standard scheme seems to not support more favorable results [16,17], we investigated the outcomes of the elderly population that have been hospitalized due to Omicron infection, in order to observe the progression of the disease and find the risk factors that are held accountable for the stagnant recovery rate, as well as protective factors that can prevent the disease from occurring in the first place. Thus, this systematic review aims to examine the clinical outcomes of hospitalized patients aged 65 or above that carry the SARS-CoV-2 Omicron virus based on their COVID-19 vaccination status. 

## 2. Materials and Methods

**Figure 1:**
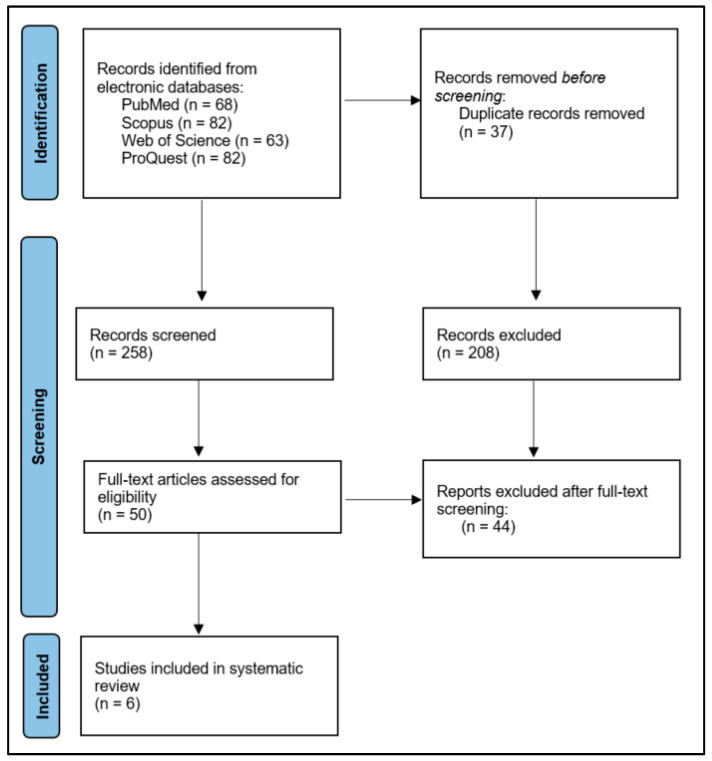
PRISMA Flow Diagram.

This systematic review used the Preferred Reporting Items for Systematic Reviews (PRISMA) guidelines [18], and PROSPERO guidelines, being registered in the Open Science Framework (OSF) platform. The phases that have been followed in this review are illustrated in Figure 1.

When conducting this research, we used the following key terms: elderly OR old OR geriatric OR senior AND omicron AND hospital* AND outcome OR effect OR consequence. In order to narrow down the results, terms referring to cohort and virus type had to be mentioned in the abstract. In order for the studies to be included, they needed to involve participants over the age of 65 who had been hospitalized. Additionally, the study group needed to be infected with an Omicron variant infection, and the article needed to record the clinical outcomes of the virus. Despite the fact that all of the collected articles were published in English, there were no further search limitations.

This study was conducted using four electronic databases for the duration of the COVID-19 pandemic, with a search query from January 2020 to December 2022, namely PubMed, Scopus, Web of Science, and ProQuest Central. Initial results from the search yielded 295 articles, of which 37 were duplicates. After eliminating 208 abstract-screened articles, we evaluated 50 full-text articles, from which we selected six for inclusion in this systematic review. The main reasons for exclusions were (1) the study cohort had less than 65 years, (2) the data extracted included people that were not hospitalized, and (3) there was a lack of statistics on clinical outcomes after becoming infected with the Omicron variant. To analyze the differences between vaccinated and unvaccinated patients, Student’s t-test was used to compare the duration of hospitalization and the Chi-square test was used to compare the proportions of patients necessitating mechanical ventilation and the mortality rate. The level of significance was considered for *p*-values < 0.05. 

Utilizing the NHLBI-published Study Quality Assessment Tools, two researchers (V.B. and F.B.) evaluated information from published articles and reported results individually. The tools are unique to research designs and screen for any methodological or operational problems. The Quality Assessment Tool for Observational Cohort and Cross-Sectional Investigations was used for the remaining studies [19]. For each of a tool’s 14 questions, “Yes” replies were worth 1 point, while “No” and “Other” responses were worth 0 points. The final performance score was then calculated. Therefore, investigations with a rating between 0 and 4 were deemed to be of low quality, studies with a grade between 5 and 9 were deemed to be of medium quality, and studies with a grade of 10 or more were evaluated to be of good quality. In order to overcome the underlying biases of the included publications, two investigators were appointed to evaluate the quality of the chosen studies, therefore lowering the danger of selection bias, missing data, and measurement bias. 

## 3. Results

### 3.1. Study Characteristics

Articles included in this systematic review had a multicenter retrospective cohort study design [20,21,22,23,24] and a multicenter prospective cohort study design [25]. Two of the studies included took place in the USA [21,22], while the other ones took place in Israel [20], China [23], Belgium [24], and Australia [25]. The number of patients included was relatively high, ranging from 3056 [21] to 445 [24], and a total of 7398 patients were identified in all six studies. The mean age was 65 [21], 68.5 [22], 76 [23], 78 [24], and 80 [20]. Only one study did not report the mean age, mentioning only the category, specifically older than 65 [25]. There were no significant gender differences, as all included studies had an equal number of males and females. Apart from the COVID-19 Omicron infection, most of the patients enrolled in the studies had various comorbidities, such as hypertension, chronic renal failure, chronic lung disease, cancer, immunosuppression, dementia, and diabetes [20,21,22,23,24,25]. The main demographic characteristics are included in Table 1. 

### 3.2. Clinical Outcomes

**Figure 2:**
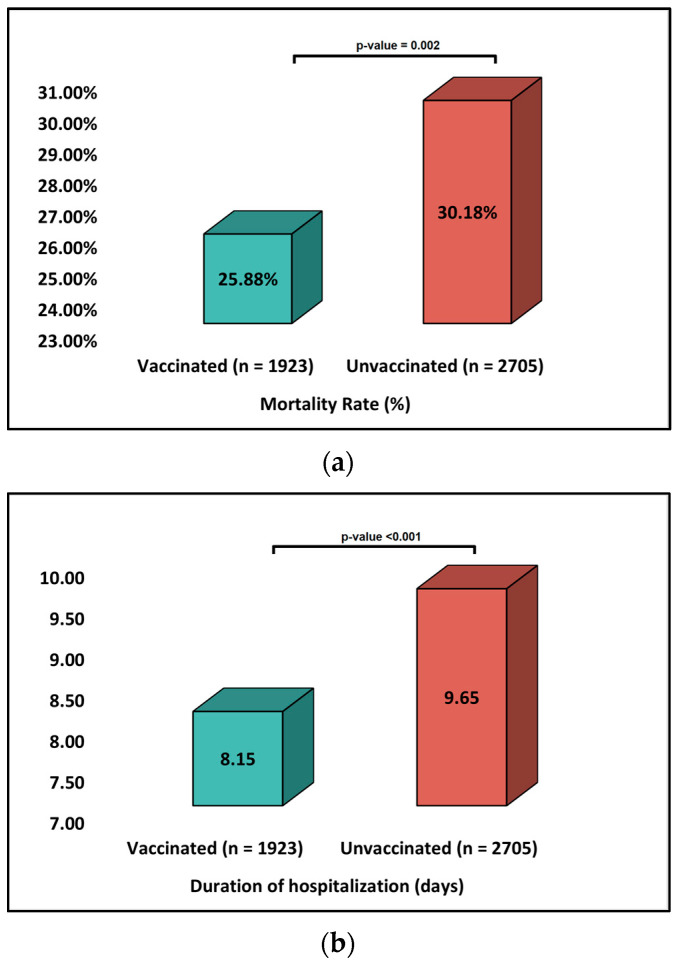

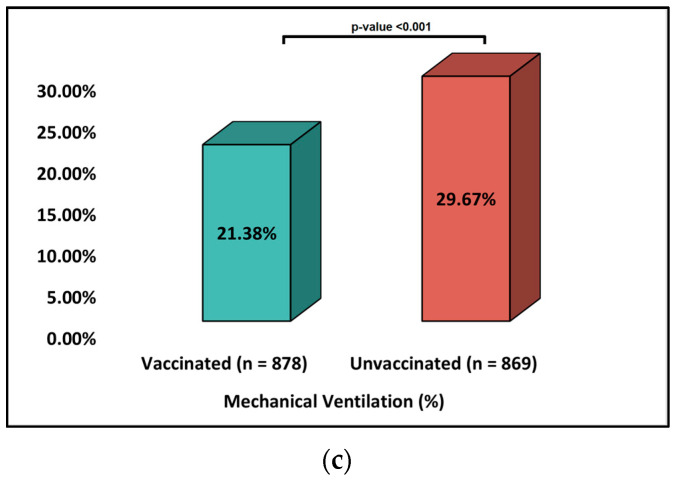
Summary of findings (vaccinated vs. unvaccinated patients); (**a**) Duration of hospitalization; (**b**) Mechanical ventilation; (**c**) Mortality Rate.

The primary outcomes analyzed were the length of hospital stay, the requirement for intensive care unit (ICU) admission, the need for mechanical ventilation, and the mortality rate, which can be seen in Table 2, and Figure 2. Two of the studies had a mean of 6 days hospitalized [20,21], while the other two had a mean of 8 days [23,24], following the most extended period, 12.5 means days [25]. One of the studies did not state the mean number of days hospitalized for the whole cohort [22]. While two studies had the total sample of the group in the intensive care unit [21,25], other than one study that did not specify the need for intensive unit care [23], other studies had a part of the sample group being in need of ICU, specifically 26.5% from the unvaccinated patients and 18.7% for fully vaccinated patients in one study [22] and 7% in another study [24]. In terms of mechanical ventilation, the proportion of patients in need was 6.7% [21], 10.6% [22], 11.6% [23], and 1.8% [24]. The mortality rate ranged from 0.51% to 20.1%, having only one study with a high mortality rate, 47% from a group sample with severe COVID-19 Omicron type [1]. 

Four of the studies evaluated clinical outcomes by dividing the study group into unvaccinated and vaccinated patients. One study found that when comparing the unvaccinated patients to those who had a three-dose vaccine, there were no differences in the rates of mortality or the need for mechanical ventilation, with both groups having a similar risk for a poor outcome [20]. When comparing to a group that had a four-dose vaccine, the mortality rates went significantly lower for the four-dose patients, having a rate of 34%, compared to the unvaccinated patients, that had a rate of 51%, in this degree is a protective factor against poor outcomes [20]. Two studies that took place in the USA had the same results, concluding that there are no differences in mortality rates between the vaccinated and unvaccinated patients, with mechanical ventilation being the first factor that raises the mortality rate [21,22], though one of the studies found that unvaccinated patients required supplemental oxygen and underlined the fact that patients that have multiple booster doses are either the oldest ones or the severely immunosuppressed [22]. Given this, even though the mortality rates for each group remain the same, unvaccinated patients have fewer comorbidities, whereas boosted patients are part of the vulnerable population when it comes to other conditions. 

One study identified that 75.9% of all patients had at least one comorbidity, with hypertension (57.9%), diabetes (23.5%), and cardiovascular disease (20.7%) being the most common, with this being one of the factors that progresses the disease’s severe outcomes, followed by being over 80 years old [23]. Despite this, having the vaccine appeared to act as a protective factor against severe outcomes, having a critical rate of 2.4%, versus 8.37% for the unvaccinated patients [23]. A different study indicated the presence of comorbidities among patients with Omicron and of older age and emphasized that compared to the Delta variant, the Omicron patients had a lower risk of severe COVID-19 and ICU admission, although there was not a significant statistical difference for the risk of death [24]. The last study included found that vaccinated patients had more comorbidities, the most prevalent being diabetes, chronic cardiac failure, chronic pulmonary disease, and immunosuppression and that patients who received three doses of immunizations were less likely to need invasive ventilation and tracheostomy than those who received only one or two doses [25]. 

Additionally, the vaccinated group had a shorter duration of ventilation and length of the hospital of ICU stay than the unvaccinated group, although there was no statistically significant association between vaccination and mortality rate [25]. All studies included required ICU-level care [20,21,22,23,24,25]. Considering the need for mechanical ventilation, three of the studies found that there was not a significant difference between the vaccinated and unvaccinated groups, with the proportion of patients in need remaining relatively the same [20,21,22,23,24,25]. The only noticeable difference was when comparing unvaccinated patients with patients that have received more than two doses of vaccine, the proportion dropping from 20% to 16% [20], although another study had a lower proportion of people in need of mechanical ventilation in the two-dose group (8.6%), compared to the boosted group (10.3%) [22]. Table 3 summarizes clinical outcomes for vaccinated and unvaccinated patients.

## 4. Discussion

The rapid spread of the Omicron variant, along with its exceedingly high infectiousness, led to an increased need for hospital admissions, questioning the effectiveness of the vaccine for certain categories of infected people [7,26]. The present study concluded, in line with other systematic reviews [7,8], that although the Omicron wave had milder effects on the population, the hospital admission rates, as well as the mortality rates, remained high, particularly among the elderly population. Although there was a lack of statistical evidence that could differentiate negative outcomes between waves, all of the studies that were included in this systematic review highlighted the fact that although it appeared as though there were no differences between people who were unvaccinated and vaccinated people, it is extremely important to look at the baseline characteristics of the patients [20,21,22,23,24,25]. Vaccinated patients who were either hospitalized or admitted to an ICU constituted the oldest segment of the population and had a series of comorbidities that acted as risk factors in developing a severe form of the disease [20,22,23,24,25]. In conclusion, patients that were either hospitalized or admitted to an ICU were given the highest risk of developing a severe form of the disease, although most were vaccinated up to date. Every study included in this review had either the whole cohort or a segment of it in the ICU [20,21,22,23,24,25], while four of them stated the need for mechanical ventilation [21,22,23,24]. A part of the studies included found that when using a third or fourth dose as a booster vaccine, the hospitalization and mortality rates dropped considerably [20,26]. Other studies stress the effectiveness of a booster dose for the older population, as it has been found to be a protective factor in developing severe symptoms, although the efficiency isn’t as high as it should be [11,27]. Aside from vaccination, it remains important to use a series of physical preventive measures such as mask use, regular ventilation, physical distance, and hand hygiene [28,29].

An expert review concluded that the efficacy of vaccines continues to prevent severe illness, hospital admission, and mortality [17]. This conclusion was reached regardless of the type of vaccine that was used [17]. In order to increase its effectiveness in combating highly infectious variations such as Omicron, it is essential to decrease the amount of time that passes between booster shots for the population that is most susceptible to infection [17,21,30]. Although booster shots appear to be the only protective option that has been found to be effective, there are a series of impediments when applying this treatment scheme. For example, low-resource countries do not have vaccines for their population; as a result, this treatment option could not reach the global population that was found to be at risk [31].

This study has a number of limitations, despite the fact that the conclusions that were drawn are consistent with the existing body of research. These conclusions concern the severe clinical outcomes that the elderly population that is infected with COVID-19 suffers, as well as the fact that the vaccine is not a powerful enough protective factor. Firstly, there were fewer publications available, which made it more difficult to extrapolate a general conclusion. Second, because the research was carried out only in the United States of America, Israel, Belgium, Australia, and China [20,21,22,23,24,25], we are unable to discuss the current state of events in any other countries. According to the findings of one study, there are billions of people who have not been vaccinated due to a lack of vaccine doses. As a result, their clinical outcomes may be more severe than the rates that we have observed [31]. The group sample, although appearing to be high, it is relatively low when compared to the number of infected people globally; therefore, the study sample size may represent another limitation. 

When it came to the reporting of statistical data on clinical outcomes, only a portion of the studies had documented clinical outcomes comparing the unvaccinated cohort with the vaccinated one [20,21,22,25]. This was done in order to determine whether or not the vaccine was effective. Although the rates of mortality and hospitalization remain high between groups, it is important to differentiate between the vaccinated and the unvaccinated people because the two types of cohorts do not share the same characteristics. The vaccinated people that are infected are part of the more vulnerable population because of the comorbidities that interfere with the virus evolution [7]. To the same extent, none of the studies took into consideration the type of vaccine used. Since there are currently quite a few vaccines on the market, with the SARS-CoV-2 mRNA vaccines developed by Moderna and Pfizer/BioNTech proving to be the most effective ones so far [32], research can concentrate on particular categories of vaccines in an attempt to determine the one that is most effective on a given population. Another limitation could be linked to the lack of age stratification, as each study used an age mean when reporting the outcomes. According to the findings of a number of studies, people who are 80 years old or older have increased risks of contracting a severe form of the disease and spend a significant amount of time hospitalized [33]. As a consequence, different results may have been observed if the targeted group was stratified according to age. However, due to the fact that each of the included studies had patients who suffered from a variety of comorbidities [20,21,22,23,24,25], with the most common being diabetes, cardiovascular diseases, and renal diseases, we cannot estimate the outcomes of patients that, apart from the Omicron infection, are presented as clinically healthy and how much the presence of the comorbidities influence the severity of the virus.

The current systematic review is valuable because it focuses on a particular population, namely elderly patients who are hospitalized and, as a result, are at a greater risk of developing severe symptoms of a severe acute respiratory syndrome caused by Coronavirus 2 (SARS-CoV-2). The high mortality rates that have been reported, along with the various comorbidities that have been found to be a risk factor for the manifestation of the infection, highlight the fact that additional research needs to be done on this particular group of people for whom the conventional vaccine strategy does not appear to be as effective for the Omicron variant [26,30,31]. According to the findings of one study [34], the use of Molnupiravir on hospitalized patients aged over 60 was associated with a reduction in the likelihood of more severe symptoms and a shorter median length of stay in the hospital. According to the findings of other studies, Paxlovid improves symptoms associated with the infection and shortens the amount of time it takes to receive negative test results [35,36]. 

Although some studies suggest that using a booster dose will help in decreasing the risk of developing severe disease and, therefore, shorten the number of days spent hospitalized or in the ICU, it is recommended that results be evaluated carefully, as the effectiveness of the vaccines decreases in time, especially for the vulnerable population [33,37]. Taking this into consideration, future research could concentrate on developing other ways of disease prevention in addition to the already existing vaccines, which have a relatively poor success rate when applied to an aging population that is afflicted with a variety of illnesses. In addition to this, potential future research can investigate several types of risk variables, which have been demonstrated to be highly related to an unfavorable course of the disease, such as age, the presence of comorbidities, as well as mechanical ventilation [7,8,26,28]. Lastly, more overall research that targets vulnerable groups needs to be addressed, as this segment requires more mobilization in prevention and treatment [17], which is why it is essential to have a solid understanding of the disease’s mechanisms, risk, and protective factors, and the treatment methods that are the most appropriate.

## 5. Conclusions

The pandemic that has been caused by the spreading of Coronavirus and its mutation has left specialists with a number of challenges to overcome in terms of developing strategies for the prevention and treatment of the various ways in which viruses manifest. Despite the fact that a large number of experts have collaborated over the course of the past few years in order to collect as much information as they possibly could regarding the origins of the virus, the most effective types of vaccines, prevention, and hospital management, there are still some aspects that remain unknown. This is because the most recent wave of the pandemic, which was caused by the Omicron variant of the virus, had the highest rate of transmission. Furthermore, despite the fact that the symptoms may have seemed to be less severe, there is still a significant portion of the population that comes into contact with this virus and experiences severe problems. These problems include increased mortality rates, admissions to intensive care units, and the requirement for mechanical ventilation. 

These findings point to the necessity of finding effective treatments for the vulnerable population. The use of a series of booster vaccines is not a feasible option because there has been insufficient research done to evaluate the vaccine’s long-term effectiveness and because it is unable to reach the global population because it requires a large number of doses to be distributed all over the world.

## Figures and Tables

**Table 1:** Demographic characteristics of studies.

Study	Country	Study Group	Age (Median)	Sex (Men)
1 [20] Brosh-Nissimov, T. et al. Hospitalized Patients with severe COVID-19 during the Omicron wave in Israel—benefits of a fourth vaccine dose. *Clinical infectious diseases: an official publication of the Infectious Diseases Society of America*. **2022**. ciac501. Advanced online publication.	Israel	877	80	51%
2 [21] Tandon, P. et al. The fourth wave: vaccination status and intensive care unit mortality at a large hospital system in New York City. *Acute and critical care*, **2022** 37(3), 339–346.	USA	3056	65	49%
3 [22] Moffa, M. A. et al. Description of Hospitalizations due to the Severe Acute Respiratory Syndrome Coronavirus 2 Omicron Variant Based on Vaccination Status. *Open forum infectious diseases*, **2022** 9(9), ofac438.	USA	667	68.5	47.8%
4 [23] Lu, G. et al. Geriatric risk and protective factors for serious COVID-19 outcomes among older adults in Shanghai Omicron wave. *Emerging microbes and infections*, **2022** 11(1), 2045–2054.	China	1377	76	46.6%
5 [24] Van Goethem, N. et al. Clinical Severity of SARS-CoV-2 Omicron Variant Compared with Delta among Hospitalized COVID-19 Patients in Belgium during Autumn and Winter Season 2021–2022. *Viruses*. **2022**, 14, 1297.	Belgium	445	78	62.2%
6 [25] Otto, M. Clinical Characteristics and Outcomes of Critically Ill Patients with 1, 2, and 3 doses of Vaccination against COVID-19 in Australia. *Internal medicine journal*, **2022**	Australia	976	>65	63.8%

**Table 2:** Clinical outcomes.

Study	Duration of Hospitalization (Median)	Mechanical Ventilation	Mortality Rate	Comorbidities
1 [20]	6	NR	47%	Hypertension, BMI > 30, chronic renal failure, cancer, immunosuppression
2 [21]	6	6.7%	9.9%	Diabetes, cardiovascular disease
3 [22]	NR	10.6%	11.3%	Immunosuppression, cardiovascular disease, chronic kidney disease, chronic lung disease, cancer, dementia
4 [23]	8	11.6%	0.51%	Cardiovascular disease, chronic kidney disease
5 [24]	8	1.8%	11.6%	Cardiovascular disease, chronic renal disease, chronic cognitive deficit, chronic immunosuppression, chronic neurological disease, obesity
6 [25]	12.5	NR	20.1%	Diabetes, obesity, chronic cardiac failure, chronic pulmonary disease, immunosuppression, chronic kidney disease, cancer

NR—Not Reported.

**Table 3:** Clinical outcomes by vaccination status.

Study	Vaccinated					Unvaccinated			
Study	Number of Doses	Required ICU-Level Care	Duration of Hospital Stay (Median)	Mechanic Ventilation	Mortality Rate	Required ICU-Level Care	Duration of Hospital Stay (Median)	Mechanic Ventilation	Mortality Rate
[20]	3 (n = 386)	Yes	6	22%	44%	Yes	6 (n = 394)	20%	47%
	4 (n = 88)	Yes	5	16%	30%				
[21]	3 (n = 88)	Yes	13	50%	38%	Yes	15 (n = 124)	57%	45%
[22]	2 (n = 209)	Yes	3.6	8.6%	10.5%	Yes	4.2 (n = 351)	12%	12.3%
	3 (n = 107)	Yes	4	10.3%	10.3%				
[25]	1 (n = 430)	Yes	13.6	Yes	21.9%	Yes	13.4 (n = 1836)	Yes	16.4%
	2 (n = 671)	Yes	10.6	Yes	25.3%				
	3 (n = 86)	Yes	6.6	Yes	27.0%				

## Data Availability

Not applicable.
